# Developmental Stages Affect the Capacity to Produce Aldehyde Green Leaf Volatiles in *Zea mays* and *Vigna radiata*

**DOI:** 10.3390/plants11040526

**Published:** 2022-02-15

**Authors:** Jurgen Engelberth, Marie Engelberth

**Affiliations:** Department of Integrative Biology, The University of Texas at San Antonio, One UTSA Circle, San Antonio, TX 78249, USA; marie.engelberth@utsa.edu

**Keywords:** green leaf volatiles, biosynthesis, tissues, development

## Abstract

Green leaf volatiles (GLV) are essentially produced by the green parts of plants upon damage. GLV are mainly 6-carbon molecules derived from fatty acids through the hydroperoxide lyase pathway and can serve as airborne signals to other parts of the same plant and to neighboring plants and help to protect them against biotic and abiotic stresses. However, while the biosynthesis is generally well understood, little is known about how plants regulate the production of these important signaling molecules. To better understand how the developmental stage of the plant affects aldehyde GLV production, we selected *Zea mays* and *Vigna radiata* to represent mono- and dicot plants for this analysis. We show that the capacity to produce aldehyde GLV strongly depends on the developmental stage of the plant. Major differences in the quantity, and in the quality of these compounds were found, not only in leaves from different developmental stages, but also in different areas within a leaf. The results demonstrate that the capacity to produce GLV varies significantly within a plant and the potential implications of these findings are discussed.

## 1. Introduction

Green leaf volatiles (GLV), the typical “green” smell of plants, are rapidly released by plants upon mechanical damage in large quantities, which can be caused by insect herbivore, certain pathogens, as well as by abiotic stresses including cold, heat, and drought [1,2]. The almost instant release after damage makes these compounds ideal chemical messengers, that can convey the potential for future damage to other distant parts of the same plant or to other plants nearby. There, GLV can activate specific defense responses, which can either provide direct protection, or prepare effectively against the impending potential for damage by biotic and abiotic stressors [3,4,5,6,7,8,9,10]. While this was first demonstrated for maize [10], it has since been shown for many other plants species [1,2].

The biosynthesis of these compounds is generally well understood [11,12,13,14,15] and begins with the addition of molecular oxygen to a fatty acid by a lipoxygenase (LOX) [16]. The resulting hydroperoxy fatty acid is then cleaved by the enzyme hydroperoxide lyase (HPL) to produce either (Z)-3-hexenal (Z3al) (from 18:3 fatty acids) or n-hexanal (nHal) (from 18:2 or less saturated fatty acids). Recently identified isomerases can then convert Z3al into E-2-hexenal (E2al) [16,17]. However, not all plants possess this enzyme, and while LOX and HPL are mostly localized in the chloroplast, the isomerase appears to be cytoplasmic [17]. Additionally, while all aldehydes are produced directly by the damaged plant cells, further transformation into their corresponding alcohols and esters occurs in intact living cells that are exposed to them [13,14,18,19].

Previously, we described how different plant species have very distinct capacities to produce GLV, ranging from non-detectable levels (*Tillandsia recurvata*) after mechanical damage to more than 80µg/g fresh weight in mung beans (*Vigna radiata*) [19]. While we identified a strong positive correlation between the production of E2al and the total amount of aldehyde GLV released by damaged plant tissue, we were not able to identify any other pattern in the release capacity and concluded that yet to be identified ecophysiological factors might be responsible. However, the study provided never before reported information about the differences between various plant species in their capacity to produce these semiochemicals with potential impact on the signaling strength in plant–plant interactions. Furthermore, in view of recent findings regarding the ability to suppress the production of GLV on the aldehyde level by various insect herbivores [20,21,22,23,24] correlations between the plant’s capacity to produce these compounds and the insect herbivore’s capacity to block them are essential for our understanding of how these mechanisms have developed during the co-evolution of these two groups.

However, while our previous study mainly focused on the general capacity to produce aldehyde GLV in more than 50 plant species [19], it did not include a more detailed analysis of how, for example, the environment or developmental stages affect this process. Therefore, we started by looking at how development affects the capacity to produce aldehyde GLV. We selected two model plants, *Zea mays* and *Vigna radiata* since they represent monocot and dicot plants and showed very different qualities and quantities of aldehyde GLV in our previous study.

## 2. Results

### 2.1. Zea mays

**Figure 1:**
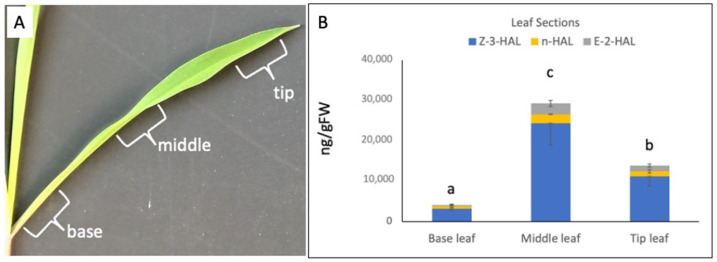
Distribution of the capacity to produce aldehyde GLV in a fully developed maize leaf at the V2 stage. (**A**) A fully developed maize leaf blade from the second leaf is shown with the segments used for this analysis. (**B**) Results for Z-3-hexenal (Z3al), n-hexanal (nHal), and E-2-hexenal (E2al) in base, middle, and tip leaf segments. The values are means ± standard deviation (SD), and the different letters indicate significant differences (ANOVA, *p* ≤ 0.05, calculated only for the total amount of aldehyde GLV).

We first tested different sections of a fully developed 2nd leaf blade from a 10-day-old maize seedling at the V2 stage (Figure 1A) to gain further insights into the general distribution of the capacity to produce GLV. At the base of the leaf blade, we found the lowest capacity to produce aldehyde GLV with 3191 ± 411 ng/gFW for Z3al and 573 ± 316 and 305 ± 175 ng/gFW for nHal and E2al, respectively (Figure 1B). Maximum levels of aldehyde GLV were found in the middle section of the leaf blade with 24,292 ± 5307 ng/gFW for Z3al, and 2220 ± 62 for nHal and 2706 ± 745 ng/gFW for E2al. At the tip of the leaf blade, the capacity decreased again significantly to 11,176 ± 2353 ng/gFW for Z3al, 1228 ± 419 ng/Fw for nHal, and 1446 ± 444 ng/gFW for E2al.

**Figure 2:**
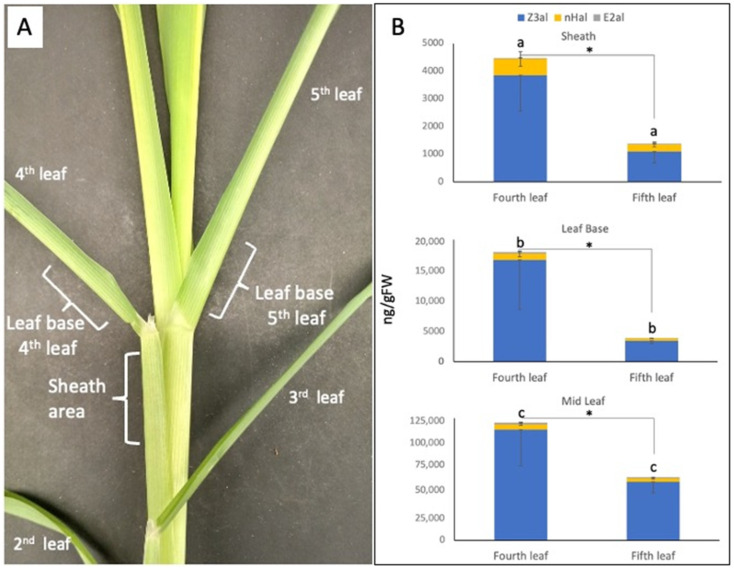
Distribution of the capacity to produce aldehyde GLV in a fully developed maize leaf. (**A**) A portion of a 25-day-old maize plant is shown with the segments used for the analysis marked. Note that the middle leaf blade segment is not shown. (**B**) Results for Z-3-hexenal (Z3al), n-hexanal (nHal), and E-2-hexenal (E2al) in the sheath, base, and mid-leaf area. The values are means ± SD, and the asterisk indicate significant differences between leaves (Total aldehyde GLV, Students *t*-test, *p* ≤ 0.05). Different letters indicate significant differences (ANOVA, *p* ≤ 0.05) in the total amount of aldehyde GLV for the comparison of segments within one leaf.

We further analyzed the base area of a maize leaf where the leaf blade converts into the sheath, which is separated from the main leaf blade by the auricle and the ligule. We selected the area just above the auricle (leaf base) as well as the sheath area just below (Figure 2A). We analyzed those areas from the 4th and the 5th leaf as well as the middle sections of the selected leaves for comparison. We used 25-day-old maize seedlings because in those plants the size of the area was large enough to allow for a clear separation without causing additional damage. For the sheath and the base area we found that the capacity for aldehyde GLV was significantly higher in the 4th leaf (16,664 ± 8090 ng/gFW Z3al, 1017 ± 482 ng/gFW nHal, and 272 ± 136 ng/gFW E2al in the leaf base area, and 3839 ± 1286 ng/gFW Z3al, 597 ± 266 ng/gFW nHal, and 40 ±10 ng/gFW E2al in the sheath area) than in the 5th leaf (3393 ± 416 ng/gFW Z3al, 429 ± 33 ng/gFW nHal, and 51 ± 18 ng/gFW E2al in the leaf base area, and 1085 ± 402 ng/gFW Z3al, 263 ± 85 ng/gFW nHal, and 27 ± 12 ng/gFW E2al in the sheath area) (Figure 2B). Furthermore, a significant difference was found again between the leaf base area and the mid-leaf section (Figure 2B). Here, we found 113,227 ±36,915 ng/gFW of Z3al, 5502 ± 1122 ng/gFW nHal, and 1942 ± 500 ng/gFW E2al in the fourth leaf, and 59,941 ± 11,335 ng/gFW of Z3al, 3657 ± 771 ng/gFW nHal, and 859 ± 208 ng/gFW E2al in the fifth leaf in the respective mid-leaf section.

**Figure 3:**
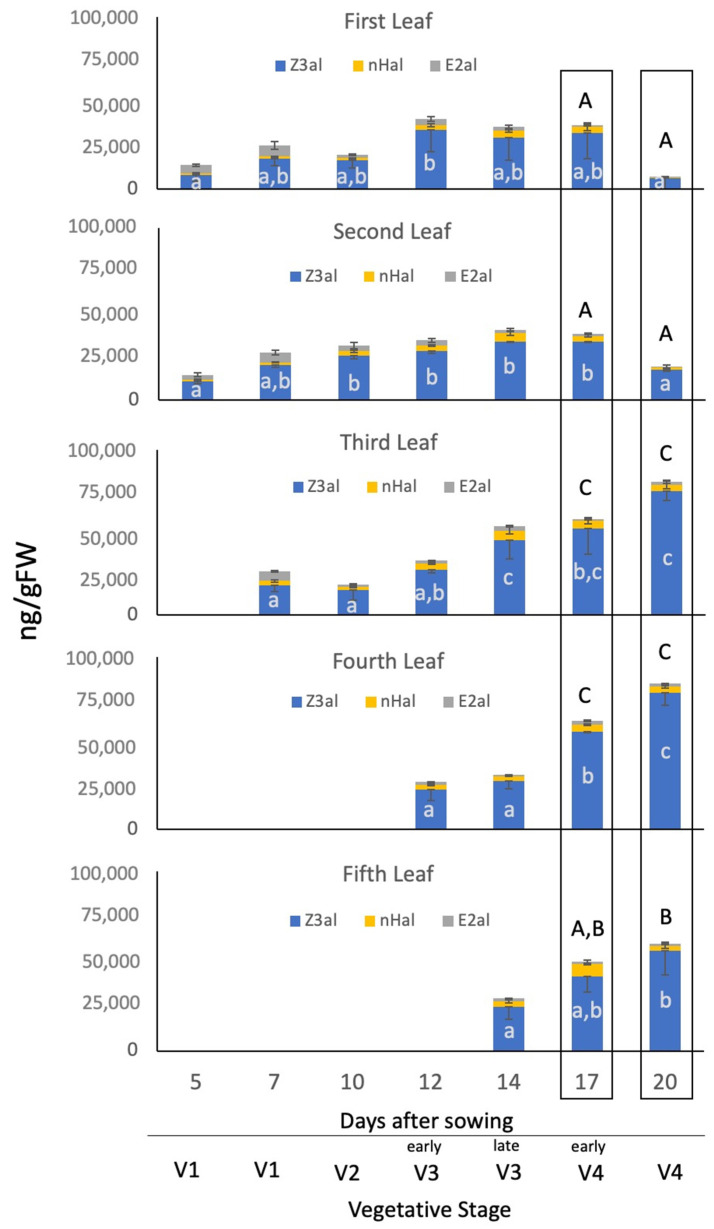
Capacity to produce aldehyde GLV at different developmental stages (V1–V4) in maize. Results for Z-3-hexenal (Z3al), n-hexanal (nHal), and E-2-hexenal (E2al) at different ages and their corresponding developmental vegetative stage are shown. The values are means ± SD, and the different letters indicate significant differences (ANOVA, *p* ≤ 0.05) in the total amount of aldehyde GLV. White letters inside the bars indicate differences within one leaf during development, while black letters above bars indicate differences between leaves (only for day 17 and 20 after sowing, marked by the framed boxes).

Since these aldehyde GLV levels in the mid-leaf segments were significantly higher than those found in 10-day-old seedlings (for comparison see Figure 1B), we performed an analysis of the mid-leaf sections during the development of a maize seedling over a period of 20 days to test for qualitative and quantitative changes during this time (Figure 3). The first analysis was performed with 5-day-old maize seedlings at the V1 stage with the second leaf still developing.

We found that the first two leaves produced very similar amounts of aldehyde GLV, in particular, Z3al (1st leaf 8471 ± 3710 ng/gFW, 2nd leaf 10,721 ± 5597 ng/gFW), but also nHal (1st leaf 937 ± 304 ng/gFW, 2nd leaf 1151 ± 453 ng/gFW), and E2al (1st leaf 4764 ± 676 ng/gFW, 2nd leaf 2805 ± 1287 ng/gFW). Both leaves reached their maximum capacity after 12 days (1st leaf 34,368 ± 12,489 ng/gFW Z3al, 2732 ± 893 ng/gFW nHal, and 3665 ± 1446 ng/gFW E2al; 2nd leaf 28,408 ± 3696 ng/gFW Z3al, 3162 ± 527 ng/gFW nHal, and 3124 ± 1089 ng/gFW E2al) and held that level until day 17, after which senescence for both leaves set in, and numbers declined significantly at day 20 (Figure 3). All following leaves (3rd, 4th, and 5th) started at similar capacities at around 25,000ng/gFW total aldehyde GLV; however, the capacity in those 3 leaves increased significantly over the next 8 days until day 20 (3rd 75,062 ± 5654 ng/gFW Z3al, 3515 ± 2347 ng/gFW nHal, and 1963 ± 931 ng/gFW E2al, 4th 79,085 ± 7095 ng/gFW Z3al, 3716 ± 665 ng/gFW nHal, and 1700 ± 247 ng/gFW E2al, and 5th 55,811 ± 13,368 ng/gFW Z3al, 2676 ± 1571 ng/gFW nHal, and 1287 ± 548 ng/gFW E2al). Overall, an increase from the first 2 leaves at day 12 to the 3 and 4th leaf at day 20 of 218% was observed for total aldehyde GLV, and the fifth leaf, while still developing, reached 158%. In the same analysis we found small, but still significant, changes in the composition of aldehyde GLV. E2al contributed more than 20% towards the total aldehyde GLV up to day 7. From then on, its portion declined, reaching only 2% in 20-day-old seedlings (Figure S1). Over the same time, numbers for nHal remained mainly constant with only minor, non-significant changes being observed.

In summary, in maize the middle sections of fully developed leaves have the highest capacity to produce aldehyde GLV, which appear to increase even further as development progresses.

### 2.2. Vigna radiata

**Figure 4:**
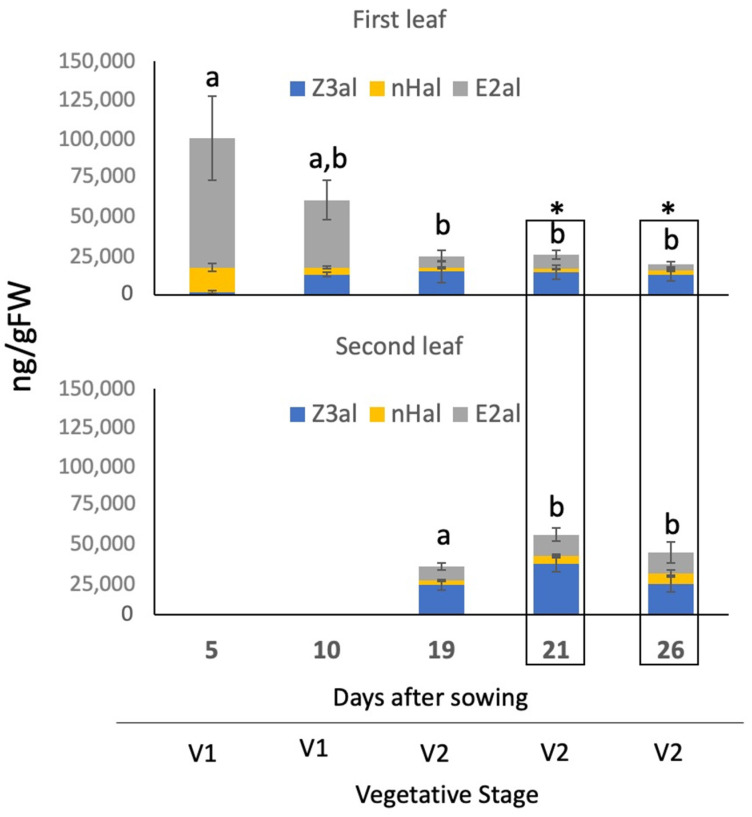
Distribution of the capacity to produce aldehyde GLV in developing mung bean (*Vigna radiata*) seedlings. Results for Z-3-hexenal (Z3al), n-hexanal (nHal), and E-2-hexenal (E2al) at different ages and their corresponding developmental vegetative stage are shown. The values are means ± SD, and different letters indicate significant differences (ANOVA, *p* ≤ 0.05) in the total amount of aldehyde GLV within one leaf. The asterisks indicate significant differences in the total amount of aldehyde GLV between the first and second leaf (Students *t*-test, *p* ≤ 0.05, marked by boxes).

*Vigna radiata* or mung bean is a dicot plant that was previously shown to emit very high quantities of aldehyde GLV with E2al being the most abundant one [20]. We started our analysis with 5-day-old plants, which had the first pair of real leaves fully developed. In those leaves we detected similar amounts when compared to our previous study [20] with 1695 ± 816 ng/gFW for Z3al, 15,761 ± 2547 ng/gFW for nHal, and 82,973 ± 26,925 ng/gFW for E2al, corresponding to 2, 16, and 82% of the emitted aldehyde GLV, respectively (Figure 4 and Figure S2). At 10 days, still at the V1 stage, we found a general decrease for all analyzed GLV, reaching only 12,996 ± 1286 ng/gFW for Z3al, 4570 ± 632 ng/gFW for nHal, and 43,159 ± 12,671 ng/gFW for E2al. Significant changes in the amount and composition of aldehyde GLV were further found in 19 days-old plants that were now at the V2 stage. Here, Z3al was the major aldehyde GLV produced by the first (14,800 ± 6929 ng/gFW) and second leaf (19,987 ± 3530 ng/gFW) corresponding to 58 and 62%, respectively, of total aldehyde GLV, while E2al was dramatically reduced (1st leaf 7500 ± 3484 ng/gFW (30%), 2nd leaf 9301 ± 2254 ng/gFW (29%)). Overall, mung bean seedlings had the lowest capacity to produce aldehyde GLV at this stage (V2 at 19 days). While the first leaf remained constant in its capacity to produced aldehyde GLV, the second leaf increased its capacity again at 21 days and 26 days after sowing with Z3al still being the major component at 33,734 ± 5178 ng/gFW at 21 days and 20,766 ± 5389 ng/gFW after 26 days corresponding to 63% and 50% of total produced aldehyde GLV (Figure S2), while E2al remained relatively low at 14,351 ± 4538 ng/gFW and 13,889 ± 6952 ng/gFW, respectively (27% and 33%).

Evidently, mung beans have the highest capacity to produce aldehyde GLV in the first pair of real leaves shortly after germination, while at later stages (V2) both the first and the second leaf, which is trifoliate, have significantly reduced capacities. Furthermore, mung beans shift during this time from a strong E2al producer to a mainly Z3al producer albeit at a much lower capacity.

## 3. Discussion

Green leaf volatiles (GLV) have now been known for more than a hundred years as chemicals that are released by plants upon damage [25,26]. For most of that time no particular function was associated to them, until about 20 years ago when it was discovered that they can provide protection for plants against insect herbivory [10,27,28]. In the following years, this was established for many different plant species and expanded to certain pathogens and more recently to abiotic stresses such as cold, heat, drought, and salinity [4,5,6,7,8,9,10,29,30,31,32,33]. Most of these studies were performed by treating plants with pure chemicals of various GLV. However, there has always been a discussion about how much of these chemicals should be used for these experiments to reflect physiological concentrations as they can occur within the plant or are released into the environment upon damage and as such can be perceived by neighboring plants, where they can elicit a variety of responses aimed to protect those against a plethora of biotic and abiotic stresses [4,5,6,7,8,9,10,27,28,29,30,31,32,33]. In our previous study, we addressed this question by showing that different plant species can produce extremely varying amounts of GLV [19]. Based on those findings, we concluded that eco-physiological factors may be the determining force behind this variety. Here, we have demonstrated that even within one plant species, the quantities but also the qualities of the produced aldehyde GLV can vary significantly during development.

In maize, the overall capacity to produce these compounds increased from the V1 to the V4 stage by nearly 500%, while in mung beans the capacity decreased significantly over the observed period. Additionally, in maize, different sections of the leaf blade varied in their capacity with the middle section of the leaf blade always having the highest capacity to produce aldehyde GLV. This information provides important data that must be taken into consideration when designing experiments aimed to study the responses of plants to different GLV. Furthermore, we previously found that plants that had the highest capacity to produce aldehyde GLV were those with an isomerase activity and suggested that in order to produce these high amounts Z3al might have to be rapidly transformed in E2al [19]. However, such an isomerase has not been identified in maize [16], but as shown herein it can nonetheless produce similar or even higher amounts purely as Z3al at later developmental stages. Since the only correlation here is between the overall size of the plant and the increased capacity to produce aldehyde GLV, the results suggest that maize plants increase their capacity in order to be able to provide sufficient quantities of GLV for effective signaling to more distant parts of the same plant should damage occur.

In mung beans, the capacity to produce aldehyde GLV differs significantly from that found in maize. Here, the first leaves were found to have the highest capacity to produce aldehyde GLV, while older leaves not only have significantly lower capacities, but also shift the quality of the produced aldehyde GLV from being an E2al emitter at very early stages to being a Z3al emitter from the V2 stage onwards. While this is somewhat similar to results described in [34] with regard to changing quantities during development, the leaves analyzed from mung beans for our study were already fully expanded and the shift was observed to correlate rather with the development of the next leaf.

At this time, we can only speculate about the reasons for this massive shift in aldehyde GLV production. In contrast to maize, where the meristem is located safely inside the sheath, the apical meristem and the axillary buds in mung beans sit openly in close proximity right between the first pair of real leaves and may, therefore, need particular protection, which can only be provided at this stage by those first two leaves that shield it. In this protection E2al may be one of the putative defense compounds and by being able to produce large quantities upon damage they may provide this protection for the otherwise unprotected meristem. Once the second leaf has been formed, mung beans possess more meristems with larger spatial separation in the form of axillary buds for additional shoot development. Therefore, the protection of a singular meristem is no longer essential for survival and the plant can reduce its protective efforts by reducing the capacity for GLV production. This is in accordance with the optimal defense theory (ODT), which states that chemical and morphological defenses of living organisms are costly, and that natural selection will favor an allocation of resources to defenses that optimize their benefit/cost ratio in terms of fitness [35,36,37]. This means defensive chemicals in plants are distributed as a function of tissue value. Since meristems are tissues of high value, they clearly fall into this category and are thus expected to be better protected by defensive chemicals, to which GLV belong.

Maize leaves in contrast seem to follow the growth-differentiation balance hypothesis (GDBH). The GDBH provides a framework that predicts a trade-off between costs of secondary metabolites relative to the demand for photosynthates by growth and predicts that outgrown plant parts will have more resources available for defense than those that are still actively growing [38,39]. In maize this clearly is the case for GLV, which, although not being secondary metabolites, are still effective defense compounds [1,2,3,4,5,6,7,8,9,10]. Fully developed leaves, as well as those at later vegetative stages with the highest photosynthesis rate, have a much higher capacity to produce these defensive compounds than those that are still growing. Therefore, maize plants at later developmental stages can invest more in defenses than younger, still growing leaves. Likewise, the base of the leaf blade and the sheath have lower photosynthesis rates and are consequently also low in their capacity to produce aldehyde.

At this time, it is unclear if this observation can be applied to certain clades of plants in general. Mung bean is a dicot plant, but no information to the best of our knowledge is available to confirm that the ODT is more likely applicable to this clade, and that the GDBH is more common for monocot plants like maize. Future research will likely clarify this point, which may have a significant impact on our approach to perform research with plants from either clade.

## 4. Materials and Methods

### 4.1. Chemicals

(*E*)-2-hexen-1-al (*E*2al), was purchased from Bedoukian (Bedoukian Research, Danbury, CT, USA). (*Z*)-3-hexen-1-al (*Z*3al), n-Hexanal (nHal), and nonyl acetate were purchased from Sigma-Aldrich (St. Louis, MO, USA). All solvents used were analytical grade.

### 4.2. Plant Material

Maize (*Zea mays*) and mung bean (*Vigna radiata*) plants were grown in Sungro Horticulture Professional Growing Mix (Sun Gro Horticulture Canada Ltd., Seba Beach, Canada) in a growth chamber under a 12 h photoperiod at 26 °C with 60% relative humidity. Light intensity was set to ca. 150 μmol m^2^ s^−1^. Plants were used at different developmental stages. For all analyses individual leaves samples from at least 3 different plants were cut, placed in a 2-mL screw cap vial, and immediately frozen in liq. N_2_.

### 4.3. Leaf Damage and Analysis of Green Leaf Volatile Synthesis

To analyze damage-induced aldehyde GLV, we homogenized the individual leaf samples containing approximately 15–30 mg of leaf tissue by following the general method described in [19] with a similar general setup also being used by [23,24]. In brief, Zirmil homogenizing beads (app. 1 mm diameter) were added to the frozen leaf samples, which were capped and then homogenized with in a Precellys tissue homogenizer (MO BIO Laboratories, Carlsbad, CA, USA) at 6000 shakes per minute for 25 s. The 2 mL microcentrifuge tubes were unscrewed without removing the cap and immediately dropped into a 30 mL glass container while also releasing the cap into the glass container to avoid significant losses in volatiles. The glass containers were immediately capped. Volatile emissions were collected right away from the tissue homogenate by inserting a volatile collection filter packed with 30-mg Hayesep Q absorbent (Supelco, Bellefonte, PA, USA) coupled to a vacuum at 0.3 L/min for 1h as described previously [20]. Filters were then removed and eluted with 150 µL dichloromethane and 1000 ng of internal standard (nonyl acetate) was added. The analysis of damage-induced GLV production was performed on a Varian 3900 gas chromatograph coupled to Varian Saturn 2200 mass spectrometer equipped with split–splitless capillary injector systems in electron impact mode (EI). Injection volume was 1 µL. The data collection, storage, and subsequent analysis were performed by using the Varian MS Workstation software. Helium at a constant flow rate of 1 mL/min was used as a carrier gas. The analyses of volatiles were performed on a fused silica capillary column (Equity™ 30 m × 0.25 mm inner diameter with a 0.25-µm-thick film of bonded methyl silicone). The GC was programmed as follows: 40 °C for 2 min, then at 15 °C/min to 250 °C. All of the injections were made in the split-mode (1:20 split ratio). Compounds were identified by comparison to authentic standards (retention time and fragmentation). Due to the strong co-elution of Z3al and nHal, we used a selected ion (*m*/*z* 56) for stronger separation of the two compounds within a single peak area (Figure S3). We then estimated the percentage of this ion in both compounds and used the calculated multiplication factor to determine the precise peak area. As a control we also used the calculated area for nHal and subtracted it from the total peak area for both compounds.

### 4.4. Statistical Analysis

At least 3 biological replicates were performed per plant. Averages and standard deviation (SD) were calculated for each of the analyzed leaf segments. For pairwise comparisons, Students *t*-test was used, while for multiple comparisons, one-way ANOVA and Tukey’s test were applied. Data can be found in Table S1 (average and standard deviation (SD)).

## 5. Conclusions

By analyzing the capacity to produce aldehyde GLV, we found that during development the quantities and qualities of the produce GLV can differ considerably. Maize leaves at later developmental stages produced significantly more aldehyde GLV than earlier leaves. In contrast, mung bean leaves decrease their capacity to produce these leaf aldehydes during development and also alter the quality of the produced aldehyde GLV by shifting from primarily being an E2al emitter to a predominantly Z3al emitter. Both, the ODT and the GDBH can be invoked to explain the observed results, but further research is imperative to prove this correlation.

## Data Availability

No new data were created or analyzed in this study.

## Supplementary Materials

The following are available online at https://www.mdpi.com/article/10.3390/plants11040526/s1, Figure S1: Composition of aldehyde green leaf volatiles in maize (*Zea mays*) during development. Figure S2: Composition of aldehyde green leaf volatiles in mung beans (*Vigna radiata*) during development. Figure S3: Selective ion quantification of aldehyde green leaf volatiles. Table S1: Aldehyde green leaf volatiles release by maize (*Zea mays*) and mung beans (*Vigna radiata*) during development.
