# Supplementary material for: Developmental Stages Affect the Capacity to Produce Aldehyde Green Leaf Volatiles in Zea mays and Vigna radiata
Source: Plants (Basel). 2022 Feb 15;11(4):526. doi: 10.3390/plants11040526 (PMC8875026; doi:10.3390/plants11040526)

Figure S1: Composition of aldehyde green leaf volatiles in maize (*Zea mays*) during development

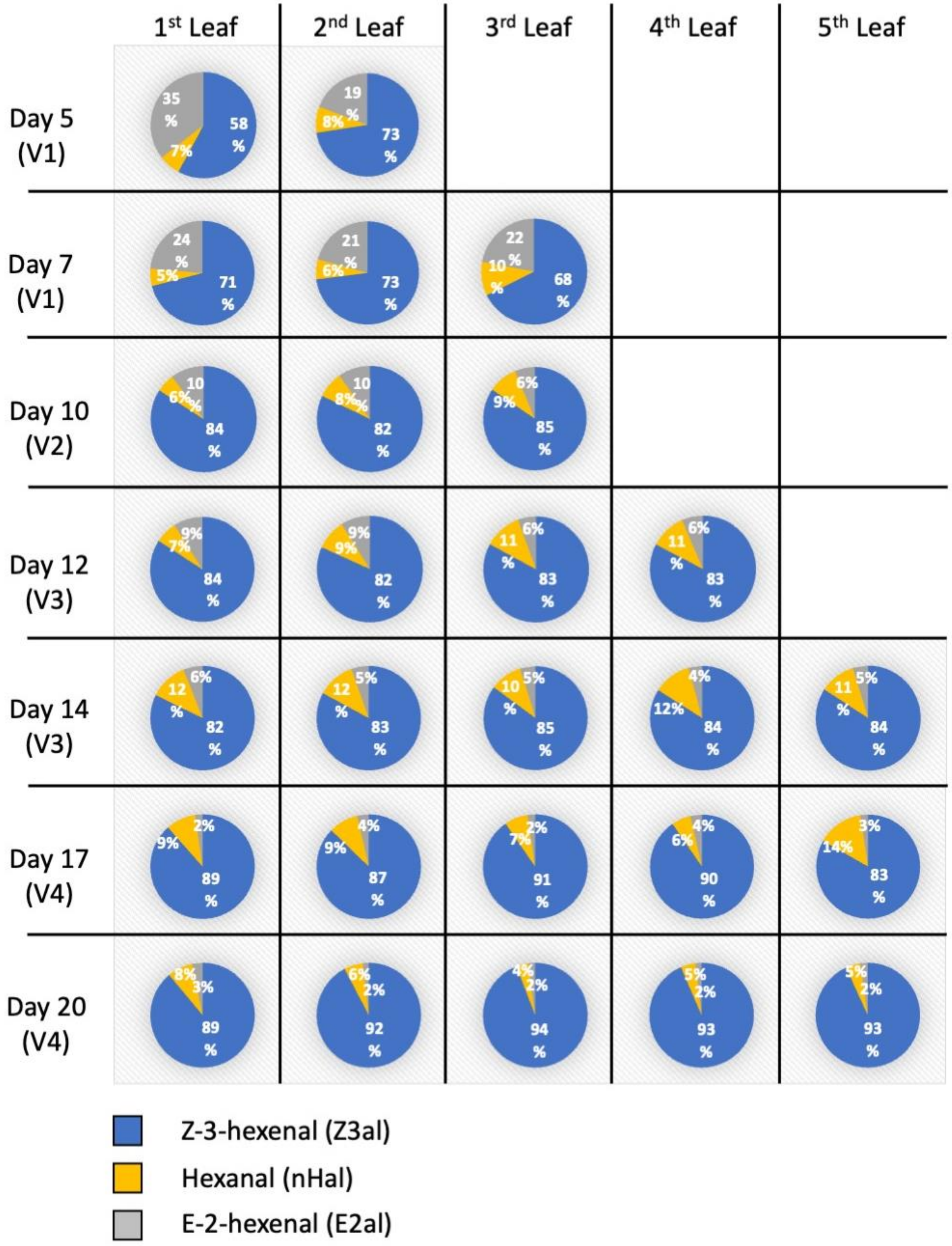

Figure S2: Composition of aldehyde green leaf volatiles in mung beans (*Vigna radiata*) during development.

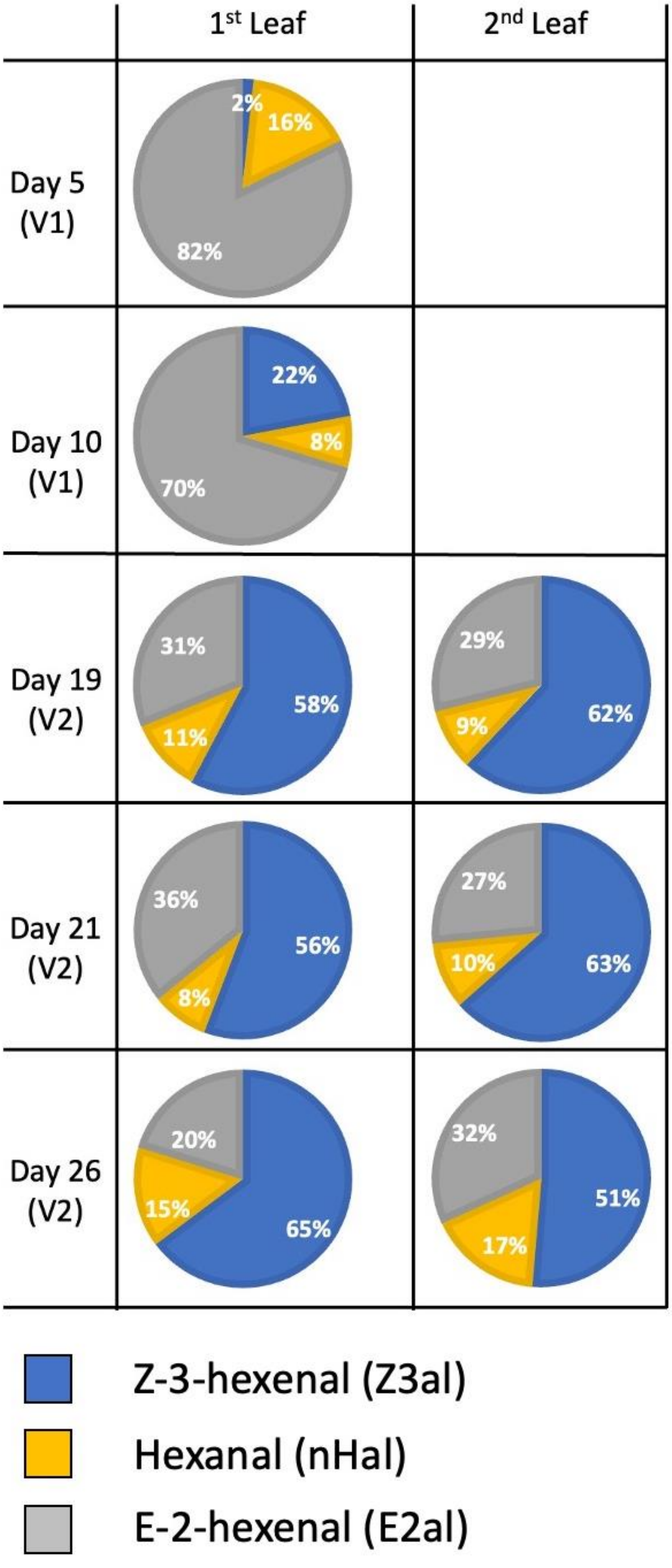

Figure S3: Selective ion quantification of aldehyde green leaf volatiles.

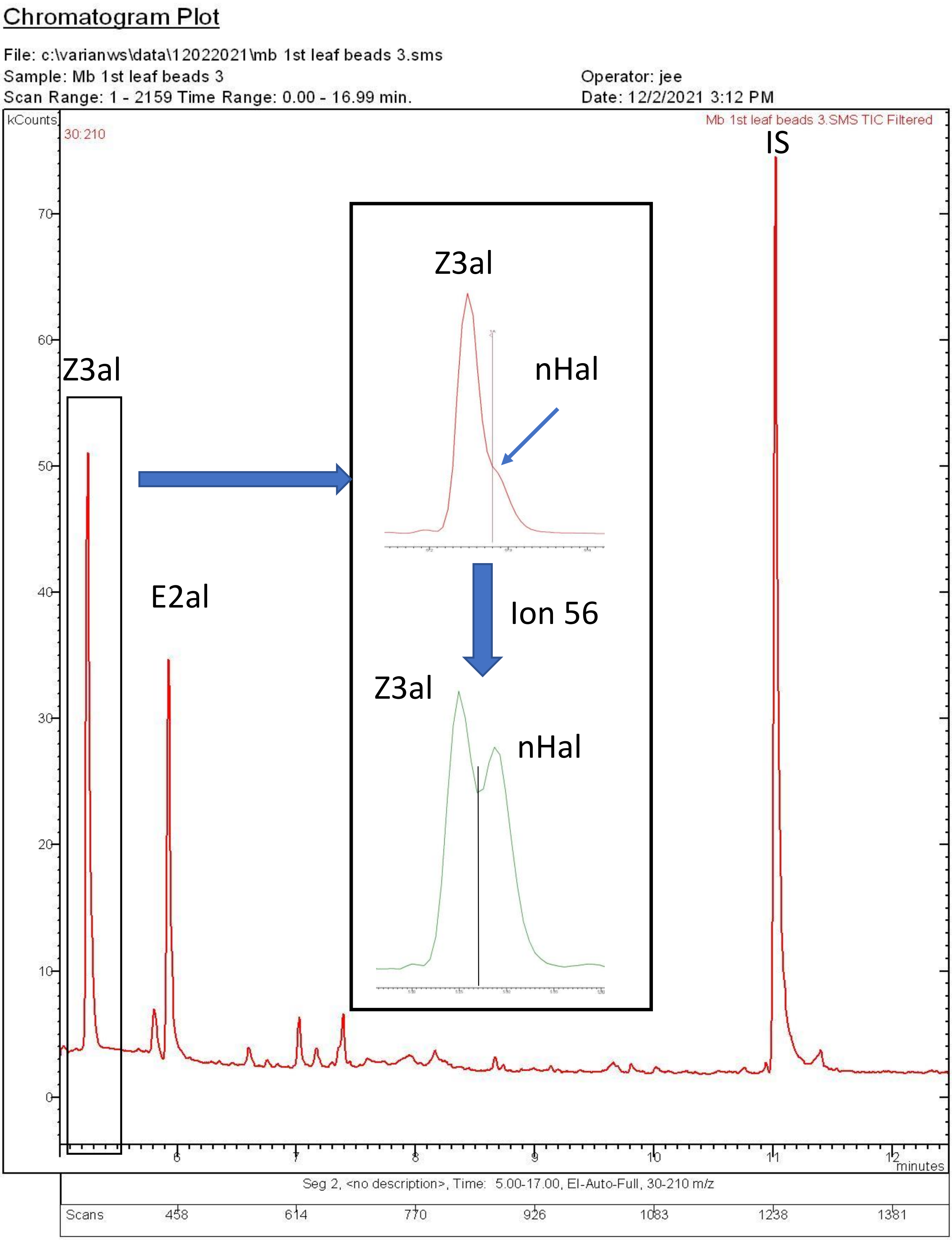

Supplement: Supplementary file 1 [file plants-11-00526-s001.zip › Supplementary Material - Figures .pdf]
