# Supplementary material for: Developmental Stages Affect the Capacity to Produce Aldehyde Green Leaf Volatiles in Zea mays and Vigna radiata
Source: Plants (Basel). 2022 Feb 15;11(4):526. doi: 10.3390/plants11040526 (PMC8875026; doi:10.3390/plants11040526)
Supplement: Supplementary file 1 [file plants-11-00526-s001.zip › Supplementary Material - Tables.pdf]

plants

MDPI

Supplementary Material

Table S1. Aldehyde green leaf volatile release by maize (*Zea mays*) and mung beans (*Vigna radiata*) during development.

Data Figure 1.

**Zea mays**						
10-days old, V2 stage	Z3al	± STDV	n-Hal	± STDV	E2al	± STDV
Base leaf blade	3,191.1	411.1	573.3	316.0	305.4	175.9
Middle leaf blade	24,292.2	5,307.3	2,220.3	62.0	2,706.9	745.2
Tip leaf blade	11,176.4	2,353.6	1,228.8	419.6	1,446.2	444.0

Data Figure 2.

**Zea mays 25-days old, V5 stage**						
Sheath	Z3al	± STDV	n-Hal	± STDV	E2al	± STDV
Fourth leaf	3,839.1	1,286.9	597.9	266.7	40.5	10.7
Fifth leaf	1,085.9	402.2	263.1	85.1	27.2	12.4
Mid leaf	Z3al	± STDV	n-Hal	± STDV	E2al	± STDV
Fourth leaf	113,227.0	36,915.6	5,502.4	1,122.6	1,942.9	500.8
Fifth leaf	59,941.1	11,335.4	3,657.4	771.6	859.3	208.5
Leaf Base	Z3al	± STDV	n-Hal	± STDV	E2al	± STDV
Fourth leaf	16,664.0	8,090.3	1,017.9	482.8	272.2	136.3
Fifth leaf	3,393.6	416.4	429.0	33.9	51.2	18.8

Data Figure 4.

**Zea mays Development (all ng/gFW)**															
Vegetative State		V1		V1		V2		early V3		late V3		early V4		V4	
days after sow-ing		5	± STDV	7	± STDV	10	± STDV	12	± STDV	14	± STDV	17	± STDV	20	± STDV
Z3al	first leaf	8,471.0	3,710.7	17,702.1	4,027.2	17,047.7	4,478.2	34,368.5	12,489.5	30,162.7	13,029.3	32,846.2	14,883.8	6,442.3	8,325.6
nHal	first leaf	937.5	304.8	1,418.9	192.5	1,043.3	324.9	2,732.0	893.1	3,771.7	550.5	3,611.8	2,181.6	687.6	519.1
E2al	first leaf	4,764.1	676.4	6,343.0	2,115.4	2,039.1	324.1	3,665.2	1,446.6	2,339.5	865.9	831.9	405.8	408.2	104.6
Z3al	second leaf	10,721.3	5,597.4	20,179.5	1,893.7	25,808.0	8,202.1	28,408.1	3,696.3	34,162.6	12,608.2	34,150.7	13,157.9	17,671.5	27,067.4
nHal	second leaf	1,151.0	453.2	1,728.3	52.2	2,610.0	614.4	3,162.2	527.9	4,618.5	1,053.4	3,091.5	644.1	1,469.6	1,448.2
E2al	second leaf	2,805.7	1,287.8	5,799.0	1,294.3	3,117.9	1,850.4	3,124.1	1,089.4	2,160.2	771.6	1,208.5	363.1	437.9	924.5
Z3al	third leaf			17,943.5	3,695.7	15,309.2	6,421.0	27,393.7	1,891.4	45,519.7	11,542.7	52,570.9	15,854.7	75,062.3	5,654.2
nHal	third leaf			2,729.1	686.9	1,703.2	560.3	3,777.9	310.8	5,666.7	2,018.4	4,334.1	1,697.6	3,515.0	2,347.9
E2al	third leaf			5,750.6	536.3	1,300.3	510.9	1,837.7	142.1	2,575.6	567.6	1,381.2	538.2	1,963.2	931.9
Z3al	4th leaf							22,945.2	6,372.0	27,969.3	4,275.4	56,759.2	808.6	79,085.8	7,095.9
nHal	5th leaf							2,858.5	538.3	3,030.8	98.6	3,787.2	425.3	3,716.2	665.0
E2al	6th leaf							1,738.3	184.0	839.1	26.1	2,260.2	487.5	1,700.8	247.2
Z3al	5th leaf									24,581.3	6,928.5	41,487.9	8,892.7	55,811.4	13,368.1
nHal	6th leaf									3,151.2	867.8	6,944.0	291.6	2,676.2	1,571.4
E2al	7th leaf									1,357.8	339.7	1,242.8	752.6	1,287.9	548.6

Data Figure 4.

**Mung Bean Development (all ng/gFW)**											
Days after sowing		5	± STDV	10	± STDV	19	± STDV	21	± STDV	26	± STDV
Z3al	1st leaf	1,695.0	816.3	12,996.5	1,286.5	14,800.2	6,929.5	14,421.4	4,402.5	12,762.4	4,079.3
nHal	1st leaf	15,761.2	2,547.3	4,570.2	632.4	2,542.1	530.1	2,062.4	178.6	2,846.7	738.1
E2al	1st leaf	82,973.2	26,925.8	43,159.0	12,671.8	7,500.7	3,484.1	9,259.9	3,030.6	3,994.4	1,558.2
Z3al	2nd leaf					19,987.8	3,530.6	33,734.7	5,178.6	20,766.4	5,389.7
nHal	2nd leaf					2,917.8	176.9	5,278.7	1,171.0	6,958.6	2,318.0
E2al	2nd leaf					9,301.1	2,254.2	14,351.6	4,538.4	13,889.4	6,952.1
